# Risks of GI endoscopy in cancer patients with neutropenia and thrombocytopenia

**DOI:** 10.3389/fgstr.2026.1890947

**Published:** 2026-08-19

**Authors:** Clark Zhang, Xiaoyi Ma, Meghan Cloutier, Michael Farrell, Edward Tamale, Debduti Mukhopadhyay, Han Yu, Anoop Prabhu, Kevin Robillard

**Affiliations:** 1 Division of Gastroenterology, Hepatology and Nutrition, Jacobs School of Medicine and Biomedical Sciences, Buffalo, NY, United States; 2 Department of Biostatistics, University at Buffalo, Buffalo, NY, United States; 3 Department of Medicine, Jacobs School of Medicine and Biomedical Sciences, Buffalo, NY, United States; 4 Department of Medicine, Sisters of Charity Hospital, Buffalo, NY, United States

**Keywords:** cancer, endoscopy, neutropenia, safety, thrombocytopenia

## Abstract

**Background:**

We evaluated the safety of endoscopic procedures in cancer patients with neutropenia and/or thrombocytopenia.

**Methods:**

We collected data on patients with neutropenia and/or thrombocytopenia who underwent endoscopy between 2012 and 2022 at Roswell Park Comprehensive Cancer Center. Neutropenia was defined as absolute neutrophil count (ANC) <1500 cells/µL, and thrombocytopenia was defined as platelet count <50 x 10³/µL. The development of infectious AEs (fever within 3 days or positive blood cultures within 7 days following endoscopy) in neutropenic patients and bleeding AEs (new overt GI bleeding within 3 days following endoscopy) in thrombocytopenic patients were the primary outcome measures; 30-day mortality was a secondary outcome measure. Univariate and multivariate analyses by logistic regressions were used to evaluate for risk factors.

**Results:**

A total of 234 patients who underwent 329 endoscopic procedures were identified. In neutropenic patients, 7.8% (8/103) developed infectious AEs. In thrombocytopenic patients, 5.8% (12/208) developed bleeding AEs. Use of granulocyte colony-stimulating factor (G-CSF) was associated with increased risk of infectious AEs (OR 5.79, *p* = 0.02). The severity of neutropenia or thrombocytopenia and risk level of endoscopy were not associated with AEs. Multivariate analyses showed that poor performance status (PS) was the greatest risk factor for increased 30-day mortality (OR 4.69, *p* = <0.01).

**Conclusions:**

Endoscopy in patients with neutropenia and thrombocytopenia is relatively safe and does not lead to significant morbidity. A careful risk-benefit analysis in cancer patients with poor PS should be conducted prior to endoscopy.

## Introduction

GI endoscopy is regarded as a safe procedure with a low rate of adverse events (AEs). Risks include bleeding, infection, and bowel perforation and are rare in average risk patients. The American Society of Gastrointestinal Endoscopy (ASGE) combined the data of over 10 million colonoscopies and calculated a perforation rate of <0.06%. (1) In a separate pooled study, the ASGE calculated the rates of bleeding, perforation, and infection in diagnostic EGD to be <0.1%, <0.01%, and <0.3%, respectively. (2) AEs are relatively elevated in higher-risk procedures such as endoscopic retrograde cholangiopancreatography (ERCP), which has an estimated complication rate of 6.9%. (3).

The safety of endoscopy in high-risk groups such as those with neutropenia and thrombocytopenia is not as well established. Neutropenic patients are generally at increased risk of developing infections, while thrombocytopenic patients are at increased risk of bleeding. The ASGE does not have high quality evidence-based guidelines on antibiotic prophylaxis or platelet transfusion thresholds in neutropenic or thrombocytopenic patients, respectively. (4, 5) A segment of the population that is particularly prone to developing cytopenias is cancer patients, mostly because of chemotherapy toxicity. Cancer patients are also at an elevated risk of developing GI-related disorders such as diarrhea, cholangitis, bleeding, and graft versus host disease (GVHD). This at-risk population also often requires evaluation with diagnostic and therapeutic endoscopy.

Without the direction of well-established guidelines, there is a gap in our practice as endoscopists in evaluating the risks versus benefits of endoscopy in these high-risk patients. The aim of this study was to assess the safety of endoscopy in these patients by assessing the development of new infectious and bleeding AEs as well as associated risk factors in neutropenic and thrombocytopenic patients, respectively.

## Methods

### Study design and population

We conducted a single center, retrospective cohort study at Roswell Park Comprehensive Cancer Center in Buffalo, NY, a tertiary care cancer center and a member of the National Comprehensive Cancer Network. Inpatient procedures performed over a 10-year period between 2012 and 2022 were evaluated. The inclusion criteria were (1) age ≥18 years old and (2) absolute neutrophil count (ANC) <1500 cells/µL and/or platelet count <50 x10^3^/µL within 24 hours prior to endoscopy. Of the patients who had ANC <1500 cells/µL, those with (1) fever within 3 days before endoscopy or (2) positive blood cultures within 7 days before endoscopy were excluded. There was no exclusion criteria for patients included only based on platelet count <50 x10^3^/µL. Before data were collected, we obtained approval from Roswell Park’s institutional review board. We retrospectively reviewed data that were extracted from Roswell Park’s electronic medical record (EMR).

### Patient characteristics

We reviewed data on age, sex, severity of neutropenia, severity of thrombocytopenia, and performance status (PS) within 3 days before endoscopy. Neutropenia severity was categorized into ANC <500 cells/µL, ANC 500 to <1000 cells/µL, and ANC 1000 to <1500 cells/µL. Thrombocytopenia severity was categorized into platelet count <10 x10^3^/µL, 10 to <20 x10^3^/µL, and 20 to <50 x10^3^/µL. If patients had multiple ANC and/or platelet count values within 24 hours before endoscopy, the nadir value within that period was recorded. The Eastern Cooperative Oncology Group (ECOG) scale was used to evaluate PS, and this was categorized into ECOG 0-1 (good PS) or ECOG 2-4 (poor PS). We also reviewed history of hematopoietic stem cell transplantation (HSCT) within 6 months before endoscopy, need for blood products transfusion within 3 days before endoscopy, use of antibiotics within 3 days before endoscopy, use of granulocyte colony-stimulating factor (G-CSF) within 3 days before endoscopy, and use of immunosuppressants within 7 days before endoscopy. Blood products transfused included packed red blood cells (PRBCs), platelets, and fresh frozen plasma (FFP). Immunosuppressants included steroids, chemotherapy, and other medications (i.e., Biologics, immunotherapy, etc.).

### Endoscopic characteristics

We reviewed data on the indication for endoscopy, type of endoscopy, presence or absence of biopsies, and risk level of endoscopy. Indication for endoscopy was categorized into: GI bleeding, evaluation for GVHD, and Other indications (including abnormal imaging, anal pain, anemia, biliary obstruction, bile leak, bowel obstruction, cholangitis, endoscopic decompression, diarrhea, inflammatory bowel disease (IBD), and pancreatitis). Types of endoscopies included esophagogastroduodenoscopy (EGD), colonoscopy, flexible sigmoidoscopy (FLX), push enteroscopy (PE), ERCP, endoscopic ultrasound (EUS), and double balloon enteroscopy (DBE). Risk level of endoscopy was categorized into low-risk or high-risk from an infection or bleeding standpoint according to pre-defined ASGE guidelines (**Supplementary Table 1**). (5, 6).

### Adverse events

In neutropenic patients, the primary outcome measure was infectious AEs. This was defined as either (1) fever within 3 days following endoscopy or (2) positive blood cultures within 7 days following endoscopy. Fever was defined as a temperature ≥38°C. Blood cultures were considered positive if there were two culture sets that grew the same organism or if one of two culture sets grew an organism and there was concern for new infection (rather than contamination) upon review of corresponding progress notes.

In thrombocytopenic patients, the primary outcome measure was bleeding AEs. This was defined as new overt GI bleeding within 3 days following endoscopy. New bleeding was assessed by reviewing endoscopy reports as well as the subjective statements of daily progress notes in the EMR. Bleeding events included need for endoscopic hemostasis during initial endoscopy, coffee-ground emesis (CGE), hematemesis, melena, and hematochezia; need for repeat endoscopy due to iatrogenic bleeding was not noted.

In all patients who met the inclusion criteria, 30-day mortality was reviewed as a secondary outcome measure.

### Statistical analysis

All data were recorded as categorical variables. Fisher’s exact test was utilized for between-group comparisons. The univariate associations for each patient or endoscopic characteristic were assessed using logistic regression. Some results were excluded due to wide or infinite confidence intervals (CIs). A multiple logistic regression analysis was conducted of variables from the univariate logistic regression that had *p* values < 0.1. Results of the univariate and multivariate analyses were recorded as odds ratio (OR) with 95% CI. Statistical significance was defined as *p* < 0.05.

## Results

### Patient characteristics

We identified 234 patients who underwent 329 endoscopic procedures (**Table 1**). The median age was 58 years (range 19–87 with 57% being male). There were 104 patients with neutropenia, 210 patients with thrombocytopenia, and 80 patients with both neutropenia and thrombocytopenia. The most common neutropenia severity was ANC <500 cells/µL in 46% of patients, and the most common thrombocytopenia severity was platelet count 20 to <50 x10^3^/µL in 79% of patients. Good PS (ECOG 0-1) was recorded in 43% of patients, whereas poor PS (ECOG 2-4) was recorded in 35% of patients. We were unable to identify the ECOG score in a minority (22%) of patients.

**Table 1:** Patient characteristics.

Variable	Frequency (Percentage)	Total
Age	63 (Median)	234
Sex		234
Male	133 (56.8%)	
Female	101 (43.2%)	
ANC count		104
<500 cells/µL	48 (46.2%)	
500-<1000 cells/µL	27 (26%)	
1000-<1500 cells/µL	29 (27.9%)	
Platelet count		210
<10 x10^3^/µL	13 (6.2%)	
10-<20 x10^3^/µL	31 (14.8%)	
20-<50 x10^3^/µL	166 (79%)	
ECOG		183
0 to 1	101 (43.2%)	
2 to 4	82 (35%)	
HSCT	139 (59.4%)	234
Antibiotics	224 (95.7%)	234
Transfusions		234
PRBCs	61 (26.1%)	
Platelets	183 (78.2%)	
FFP	4 (1.7%)	
G-CSF	45 (19.2%)	234
Immunosuppressants		234
Steroids	102 (43.6%)	
Medications	138 (59%)	
Chemotherapy	25 (10.7%)	

Regarding other patient characteristics, 59% of patients received HSCT within 6 months before endoscopy. Most patients (96%) received antibiotics within 3 days before endoscopy. Nineteen percent of patients received G-CSF within 3 days before endoscopy. Non-steroidal and non-chemotherapeutic medications were the most common type of immunosuppressant used within 7 days before endoscopy (59%), followed by steroids (44%), and chemotherapy (11%). Platelets, PRBCs, and FFP were transfused within 3 days before endoscopy in 78%, 26%, and 2% of patients, respectively.

### Endoscopic characteristics

The most common indication for endoscopy was evaluation for GVHD (54%), followed by GI bleeding (24%), and Other indications (22%) (**Table 2**). Colonoscopy was performed in 74% of patients and was the most prevalent type of procedure, followed by EGD in 67% of patients. Biopsies were taken in 72% of patients. Regarding procedural risk, 89% of procedures were low-risk and 12% were high-risk based on ASGE criteria.

**Table 2:** Endoscopic characteristics.

Total (n = 234)	
Variable	Frequency (Percentage)
Indication	
Bleeding	56 (23.9%)
GVHD	126 (53.8%)
Other	52 (22.2%)
Type	
EGD	156 (66.7%)
Colonoscopy	173 (73.9%)
FLX	28 (12%)
PE	4 (1.7%)
ERCP	24 (10.3%)
EUS	8 (3.4%)
DBE	1 (0.4%)
Biopsied	
Yes	169 (72.2%)
No	65 (27.8%)
Risk Level	
High	27 (11.5%)
Low	207 (88.5%)

### Infectious adverse events

Of the 103 neutropenic patients, a total of 8 (7.8%) developed evidence of infection defined as fever within 3 days after endoscopy or positive blood cultures within 7 days after endoscopy. Of these 8 patients, 3 developed fever and 5 developed positive blood cultures. No patients had both fever and positive blood cultures.

Use of G-CSF was the only significant risk factor associated with developing infectious AEs. Four out of 18 patients (22%) who used G-CSF within 3 days before endoscopy developed an infectious AE compared to 4 out of 85 patients (4.7%) who did not use G-CSF (*p* = 0.03) (**Table 3**). On univariate and multivariate analyses, patients who received G-CSF had 5.79 times greater odds to develop an infectious AE compared to patients who did not receive G-CSF (*p* = 0.02) (**Table 4**). Notably, the severity of ANC was not found to be a significant risk factor for developing infectious AEs (**Table 3**). Other factors including PS, history of HSCT, use of immunosuppressants (limited to steroids and medications), indication for endoscopy, and type of endoscopy (limited to EGD, colonoscopy, FLX, and EUS) showed no significant associations with developing infectious AEs on univariate analyses (**Table 4**).

**Table 3:** Development of infectious AEs.

Total (n = 103)			
Variable	No Infection	Infection	p value
ANC count			
<500 cells/µL	42 (87.5%)	6 (12.5%)	0.3580
500-<1000 cells/µL	26 (96.3%)	1 (3.7%)	
1000-<1500 cells/µL	27 (96.4%)	1 (3.6%)	
G-CSF			
No	81 (95.3%)	4 (4.7%)	0.0299
Yes	14 (77.8%)	4 (22.2%)	

**Table 4:** Univariate analyses showing the association of outcome variables with infectious AEs.

Total (n = 103)		
Variable	OR	*p* value
ANC count		
<500 cells/µL	–	
500-<1000 cells/µL	0.27 (0.03, 2.36)	0.2365
1000-<1500 cells/µL	0.26 (0.03, 2.27)	0.2231
HSCT		
No	–	
Yes	1.78 (0.4, 7.85)	0.4493
G-CSF		
No	–	–
Yes	5.79 (1.29, 25.86)	0.0216*
Immunosuppressants		
Steroids		
No	–	
Yes	0.23 (0.03, 1.98)	0.1828
Medications		
No	–	
Yes	1.11 (0.26, 4.7)	0.8862
Indication		
Bleeding	–	
GVHD	0.35 (0.07, 1.7)	0.1942
Other	0.21 (0.02, 2.03)	0.1786
Type		
EGD		
No	–	
Yes	4.47 (0.53, 37.78)	0.1696
Colon		
No	–	
Yes	0.36 (0.08, 1.54)	0.1667
FLX		
No	–	
Yes	3.19 (0.56, 18.17)	0.1923
EUS		
No	–	
Yes	2.57 (0.26, 25.16)	0.4170

Some results were excluded due to wide or infinite CIs.

*Only G-CSF qualified for a multivariate analysis (*P* value < 0.1). The same OR, 95% CI, and *P* value were found on univariate and multivariate analysis of G-CSF.

### Bleeding adverse events

A total of 12 out of 208 thrombocytopenic patients (5.8%) developed a new GI bleeding event within 3 days after endoscopy.

There was no significant difference in the rate of bleeding AEs for patients with platelet counts <10 x10^3^/µL, 10 to <20 x10^3^/µL, and 20 to <50 x10^3^/µL (**Table 5**). Other factors including PS, need for blood products transfusion (limited to PRBCs), indication for endoscopy, and type of endoscopy (limited to EGD, colonoscopy, FLX, and ERCP) showed no significant associations with developing new bleeding AEs on univariate analyses (**Table 6**).

**Table 5:** Development of bleeding AEs.

Total (n = 208)			
Variable	No Bleeding	Bleeding	p value
Platelet count			
<10 x10^3^/µL	11 (84.6%)	2 (15.4%)	0.2425
10-<20 x10^3^/µL	30 (96.8%)	1 (3.2%)	
20-<50 x10^3^/µL	155 (94.5%)	9 (5.5%)	

**Table 6:** Univariate analyses showing the association of outcome variables with bleeding AEs.

Total (n = 208)		
Variable	OR	*p* value
Platelet count		
<10 x10^3^/µL	–	
10-<20 x10^3^/µL	0.18 (0.02, 2.23)	0.1832
20-<50 x10^3^/µL	0.32 (0.06, 1.66)	0.1751
Transfusions		
PRBCs		
No	–	
Yes	1.98 (0.6, 6.51)	0.2615
Indication		
Bleeding	–	
GVHD	1.85 (0.38, 9.05)	0.4449
Other	1.46 (0.2, 10.84)	0.7131
Type		
EGD		
No	–	
Yes	0.59 (0.18, 1.93)	0.3816
Colon		
No	–	
Yes	0.92 (0.24, 3.54)	0.9035
FLX		
No	–	
Yes	1.43 (0.3, 6.94)	0.6546
ERCP		
No	–	
Yes	1.28 (0.15, 10.69)	0.8199

Some results were excluded due to wide or infinite CIs.

### 30-day mortality

Within 30 days after endoscopy, mortality was observed in 43 out of 234 patients (18%).

On univariate analyses, factors associated with increased 30-day mortality included poor PS (OR 4.47, *p* = <0.01), need for PRBCs transfusion (OR 4.04, *p* = <0.01), need for platelets transfusion (OR 4.19, *p* = 0.02), and use of steroids (OR 3.42, *p* = <0.01); whereas factors associated with decreased mortality rate included evaluation for GVHD indication for endoscopy (OR 0.31, *p* = <0.01) and Other indication for endoscopy (OR 0.11, *p* = <0.01). All other factors, including neutropenia and thrombocytopenia severity, were not significantly associated with increased 30-day mortality (**Table 7**). On multivariate analyses, only poor PS (OR 4.69, *p* = <0.01), need for PRBCs transfusion (OR 3.53, *p* = 0.01), and use of steroids (OR 2.81, *p* = 0.03) were significantly associated with increased 30-day mortality (**Table 8**).

**Table 7:** Univariate analyses showing the association of outcome variables with 30-day mortality.

Variable	OR	p value	Total
Sex			230
F	–		
M	1.54 (0.76, 3.13)	0.2289	
ANC			103
<500 cells/µL	–		
500-<1000 cells/µL	0.56 (0.1, 2.99)	0.4975	
1000-<1500 cells/µL	1.17 (0.3, 4.55)	0.8243	
Platelet count			206
<10 x10^3^/µL	–		
10-<20 x10^3^/µL	0.64 (0.13, 3.2)	0.5874	
20-<50 x10^3^/µL	0.85 (0.22, 3.28)	0.8165	
ECOG			183
0-1	–		
2-4	4.47 (2.01, 9.93)	0.0002	
HSCT			230
No	–		
Yes	0.95 (0.48, 1.89)	0.8823	
Transfusions			230
PRBCs			
No	–		
Yes	4.04 (1.99, 8.19)	0.0001	
Platelets			
No	–		
Yes	4.19 (1.24, 14.21)	0.0214	
FFP			
No	–		
Yes	4.79 (0.66, 35.08)	0.1226	
GCSF			230
No	–		
Yes	1.24 (0.54, 2.83)	0.6129	
Immunosuppressants			230
Steroids			
No	–		
Yes	3.42 (1.67, 7.03)	0.0008	
Medications			
No	–		
Yes	2.09 (0.99, 4.42)	0.0535	
Chemotherapy			
No	–		
Yes	1.53 (0.57, 4.12)	0.3958	
Indication			230
Bleeding	–		
GVHD	0.31 (0.15, 0.64)	0.0017	
Other	0.11 (0.03, 0.42)	0.0010	
Type			230
EGD			
No	–		
Yes	0.81 (0.4, 1.63)	0.5495	
Colon			
No	–		
Yes	1.57 (0.68, 3.61)	0.2931	
FLX			
No	–		
Yes	1.65 (0.65, 4.18)	0.2937	
PE			
No	–		
Yes	1.55 (0.16, 15.29)	0.7074	
EUS			
No	–		
Yes	0.65 (0.08, 5.43)	0.6909	
Biopsied			230
No	–		
Yes	1.04 (0.48, 2.22)	0.9291	

Some results were excluded due to a small number of events or because all observations at certain levels of the independent variable were negative.

**Table 8:** Multivariate analyses showing the association of outcome variables with 30-day mortality.

Variable	OR	p value
PS		
ECOG 2-4	4.69 (1.9, 11.54)	0.0008
Transfusions		
PRBCs	3.53 (1.35, 9.24)	0.0101
Platelets	4.76 (0.86, 26.5)	0.0747
Immunosuppressants		
Steroids	2.81 (1.09, 7.21)	0.0317
Medications	1.19 (0.38, 3.71)	0.7704
Indication		
GVHD	0.59 (0.2, 1.69)	0.3230
Other	0.4 (0.09, 1.91)	0.2531

## Discussion

In this retrospective study of 234 cancer patients with neutropenia and/or thrombocytopenia, 7.8% of neutropenic patients developed infectious AEs and 5.8% of thrombocytopenic patients developed bleeding AEs. The incidences of infectious and bleeding AEs were comparable to that of previous studies per our literature review. A retrospective study of 588 cancer patients by Abu-Sbeih et al. identified a 4.1% infection rate and a 4.9% bleeding rate within 1 week of endoscopy. (7) In Krishna et al., 395 patients with thrombocytopenia were evaluated for risk of bleeding AEs with routine endoscopy. The incidence of bleeding with standard forceps biopsy and polypectomy were 1.5% and 4%, respectively. (8) It is reasonable to interpret that endoscopy in neutropenic and thrombocytopenic patients is relatively safe with low morbidity. However, these rates are still higher by at least an order of magnitude compared to the infection and bleeding rates in average risk patients. According to a 2022 systematic review of 117 articles by Deb et al., the composite infection rate was 0.2% following GI endoscopic procedures. (9) A 2001 prospective study among German gastroenterologists found rates of significant hemorrhage following EGD and colonoscopy to be as low as 0.002% and 0.001%, respectively. (10).

Our study showed that infectious or bleeding AEs were independent of severity of cytopenia, PS, indication for endoscopy, type of endoscopy, and risk level of endoscopy. Infection risk in neutropenic patients was independent of history of HSCT, use of antibiotics, and use of immunosuppressants. Bleeding risk in thrombocytopenic patients was independent of need for blood products transfusion. Prior studies revealed similar findings. A study of 479 patients with neutropenia and hematologic diseases by Shin et al. found that the severity of neutropenia and the invasiveness of procedure were not associated with the development of new infectious AEs. (11) Abu Sbeih et al. noted that neutropenia severity and infectious AEs were not associated. (7) Krishna et al. found that thrombocytopenia severity was not associated with increased risk of bleeding. (8) Of note, most patients received antibiotics prior to endoscopy, which may potentially explain the lack of difference in degree of neutropenia and development of infections as antibiotic use could have prevented the development of infections.

Use of G-CSF increased the risk of developing infectious AEs, and this was the only outcome variable that showed any association with AEs. G-CSF is a glycoprotein that can significantly increase the number of white blood cells (WBCs) via augmenting the proliferation of granulopoiesis in the bone marrow; thus, its association with increased risk of infection may seem paradoxical. (12) A possible explanation for this can be derived from the controversy in the role of G-CSF in neutropenic enterocolitis. Some researchers speculate that bowel wall integrity may be impaired from the increased inflammatory response associated with myeloid reconstitution. (13) Another consideration is that patients who received G-CSF may have been higher risk to begin with as this medication is typically only reserved for severe neutropenia (ANC <500 cells/µL); however, neutropenia severity was not found to be associated with infectious AEs. It is also possible that G-CSF may have caused non-infectious fever as a side effect.

Multivariate analyses showed that poor PS, PRBCs transfusion, and use of steroids were associated with increased 30-day mortality rate, with poor PS showing the most statistical significance. Patients with poor PS had close to 5 times greater odds to die within 30-days compared to patients with good PS (*p* = <0.01). Abu-Sbeih et al. also found that poor PS was associated with increased risk of 30-day mortality. (8) Thus, a higher level of caution should be utilized during endoscopic evaluation in neutropenic and thrombocytopenic cancer patients with poor PS.

One of the strengths of our study is that extracting data from a National Comprehensive Cancer Network hospital provided us with a large sample size of patients with cytopenias who underwent GI endoscopy. Second, we reviewed multiple patient and endoscopic characteristics to account for possible confounding variables, raising the internal validity of our study.

There are also limitations to our study. First, the retrospective nature of the study does not allow us to establish causality. We can only infer that infectious or bleeding AEs were a consequence of endoscopy. It should be noted that our definition of an infectious AE as fever or positive blood cultures after endoscopy is ultimately a subjective one; for example, fevers are not always caused by infections and sometimes have non-infectious etiologies. Similarly, we can only infer that increased 30-day mortality was reflective of expected patient prognosis rather than safety of procedure due to the positive association with poor PS and lack of significant association with cytopenias severity. Additional patient characteristics such as type of cancer or clinical stage of cancer could have been recorded to further support this theory. Second, the oncology population is generally at higher risk for infection in the nosocomial setting, even without neutropenia. This limits the generalizability of our study to the average-risk patient population. Third, we were unable to record bleeding AEs objectively. We did not record the need for blood products transfusion post endoscopy as a bleeding AE because the indication for transfusion could have been not attributable to bleeding. Reviewing the subjective statements of daily progress notes for new bleeding events could have led to underreporting. Fourth, close to 24% of patients underwent endoscopic evaluation for GI bleeding, but these patients were not excluded from the analysis of bleeding AEs in those with thrombocytopenia; this could have led to overreporting.

Overall, based on our study, endoscopy in neutropenic and thrombocytopenic patients appears safe from both an infection and bleeding standpoint. Severity of cytopenia and risk level of endoscopy were not significantly associated with AEs. Use of G-CSF was associated with increased risk for infection in neutropenic patients. Poor PS was the most significant risk factor associated with 30-day mortality in all patients. The presence of these risk factors in patients undergoing upcoming endoscopic procedures should inform the risk-benefit discussions.

## Data Availability

The raw data supporting the conclusions of this article will be made available by the authors, without undue reservation.

## Supplementary material

Supplementary Table 1 Endoscopy procedure risk stratification.
