# Supplementary material for: Risks of GI endoscopy in cancer patients with neutropenia and thrombocytopenia
Source: Front Gastroenterol (Lausanne). 2026 Aug 19;5:1890947. doi: 10.3389/fgstr.2026.1890947 (PMC13533736; doi:10.3389/fgstr.2026.1890947)
Supplement: Supplementary file 1 [file Table1.pdf]

Supplementary Table 1. Endoscopy procedure risk stratification

High-risk	Low-risk
Risk of infection	
Esophageal dilation	EGD or colonoscopy with or without biopsy
Sclerotherapy of varices	Device-assisted enteroscopy
ERCP with bile ducts obstruction	EUS with or without FNA
Risk of bleeding	
Polypectomy	Biopsy
Biliary or pancreatic sphincterotomy	ERCP without sphincterotomy
PEG or PEJ placement	EUS without FNA
Therapeutic balloon-assisted enteroscopy	Push enteroscopy and diagnostic balloon-assisted enteroscopy
EUS with FNA	Enteral stent deployment
Tumor ablation	Capsule endoscopy
Ampullary resection	Argon plasma coagulation
Cystogastrostomy	Barrett's ablation
Pneumatic or bougie dilation	
Treatment of varices	
Endoscopic hemostasis	
Endoscopic mucosal resection	
Endoscopic submucosal dissection	
